# Diabetes Mellitus and Prediabetes on Kidney Transplant Waiting List- Prevalence, Metabolic Phenotyping and Risk Stratification Approach

**DOI:** 10.1371/journal.pone.0134971

**Published:** 2015-09-23

**Authors:** Martina Guthoff, Dorothea Vosseler, Julia Langanke, Silvio Nadalin, Alfred Königsrainer, Hans-Ulrich Häring, Andreas Fritsche, Nils Heyne

**Affiliations:** 1 Dept. of Endocrinology and Diabetology, Angiology, Nephrology and Clinical Chemistry, University of Tübingen, Otfried-Müller-Str. 10, 72076, Tübingen, Germany; 2 Dept. of General-, Visceral- and Transplant Surgery, University of Tübingen, Hoppe-Seyler-Str. 3, 72076, Tübingen, Germany; GDC, GERMANY

## Abstract

**Background:**

Despite a significant prognostic impact, little is known about disturbances in glucose metabolism among kidney transplant candidates. We assess the prevalence of diabetes mellitus (DM) and prediabetes on kidney transplant waiting list, its underlying pathophysiology and propose an approach for individual risk stratification.

**Methods:**

All patients on active kidney transplant waiting list of a large European university hospital transplant center were metabolically phenotyped.

**Results:**

Of 138 patients, 76 (55%) had disturbances in glucose metabolism. 22% of patients had known DM, 3% were newly diagnosed. 30% were detected to have prediabetes. Insulin sensitivity and-secretion indices allowed for identification of underlying pathophysiology and risk factors. Age independently affected insulin secretion, resulting in a relative risk for prediabetes of 2.95 (95%CI 1.38–4.83) with a cut-off at 48 years. Body mass index independently affected insulin sensitivity as a continuous variable.

**Conclusions:**

The prevalence of DM or prediabetes on kidney transplant waiting list is as high as 55%, with more than one third of patients previously undiagnosed. Oral glucose tolerance test is mandatory to detect all patients at risk. Metabolic phenotyping allows for differentiation of underlying pathophysiology and provides a basis for early individual risk stratification and specific intervention to improve patient and allograft outcome.

## Introduction

Diabetes mellitus (DM) is an increasing health care problem with substantial impact on morbidity and mortality. DM is the most common cause of end stage renal disease [1] and limits access to transplantation, mostly due to related comorbidities [2]. In transplanted patients, DM impairs patient and allograft outcome [3] and is a major determinant of death with functioning allograft.

Prediabetes summarizes (i) impaired fasting glucose (IFG), (ii) impaired glucose tolerance (IGT) in standard oral glucose tolerance test and (iii) glycated haemoglobin A1c (HbA1c) levels between 5.7% and 6.4%. As a state of impaired glucose metabolism, however not fulfilling criteria of manifest DM, prediabetes has been shown an independent risk factor for progression to both, manifest DM or posttransplantation diabetes mellitus (PTDM) [4–7]. As a modifiable risk factor, prediabetes offers a window of opportunity for early strategic intervention aiming at the prevention of PTDM.

With an incidence of 15–30% in the first year, PTDM is an increasing problem in organ transplantation [8–10]. Similar to pre-existing diabetes mellitus, PTDM impairs patient and allograft survival [6,9]. Cole and coworkers demonstrated the impact of PTDM on long-term allograft survival to be as detrimental as acute rejection [11]. The pathomechanism of PTDM is not fully understood. In analogy to diabetes mellitus, increased insulin resistance and diminished insulin secretion are the underlying causes of PTDM [12–15], however the latter seems to be the predominant factor [16]. This is underlined by the fact, that PTDM shares several risk genes with DM, most of them involved in beta cell function [17,18].

The risk of PTDM can be modified by different strategies and pre- and periinterventional management. The individual risk is determined by glucose metabolism prior to trans-plantation. Albeit a modifiable risk factor with profound prognostic implication, only few investigations exist on the prevalence of prediabetes and diabetes in dialysis patients and recent data suggest much higher numbers than assumed [19].

In the present investigations, we assess the prevalence of diabetes mellitus and prediabetes among active kidney transplant waiting list candidates of a large university hospital transplant center in Europe. We identify underlying pathologies and risk factors and propose an approach for pre-transplant risk stratification and the development of specific and individualized strategies aiming to improve patient and allograft outcome.

## Materials and Methods

All patients on active kidney transplant waiting list at the Tübingen University Hospital Collaborative Transplant Center were evaluated. As part of their regular annual waiting list exam at the transplant center, a routine baseline lab including metabolic parameters was taken after an overnight fast, followed by a standard oral glucose tolerance test (OGTT) in patients without known DM. Appointments were made such that hemodialysis patients were seen on a non-hemodialysis day (short interval). For patients on peritoneal dialysis, dialysis regime was paused the day before at 10 p.m. in order not to alter glucose metabolism. Personal and medical data were collected prospectively by patient interview with focus on underlying disease, comorbidities, medication, time and modality of renal replacement therapy, family history of diabetes in primary relatives and—in females—history of gestational diabetes mellitus. The Institutional Ethics Committee of the Medical Faculty of Tübingen specifically approved this study (631/2012BO2) and written informed consent was obtained from all patients prior to inclusion. Investigations were conducted in accordance with the declaration of Helsinki. In a pretransplant setting, the Declaration of Istanbul does not apply. Written informed consent was obtained from all patients prior to inclusion in the study.

Baseline lab included determination of HbA1c and blood lipid levels. OGTT was performed with blood sampling at time points 0, 30, 60, 90 and 120 min for determination of glucose and insulin. Patients with known diabetes mellitus or previously unknown diabetes mellitus and fasting plasma glucose ≥ 7 mmol/l upon presentation were included in the database without OGTT, as contraindicated in this setting. Prevalence of diabetes and prediabetes was assessed according to American Diabetes Association (ADA) criteria [20]. Prediabetes was defined as fasting plasma glucose (FPG) of 5.5–6.9 mmol/l (impaired fasting glucose, IFG), 2h plasma glucose in OGTT of 7.8–11.0 mmol/l (impaired glucose tolerance, IGT) or an HbA1c level of 5.7–6.4% [20]. Plasma glucose was measured on the ADVIA 1800 clinical chemistry analyzer (hexokinase method) and insulin levels were determined on the ADVIA Centaur chemiluminescent immunoassay system (Instruments from Siemens Healthcare Diagnostics, Eschborn, Germany). HbA1c was measured by high performance liquid chromatography (Tosoh 11c 2.2 HLC-723, Tokyo, Japan)

To further characterize glucose metabolism, the following insulin sensitivity and-secretion indices were calculated: (i) Homeostatic model assessment of insulin resistance (HOMA-IR) = (glucose * insulin)/22.5 [21], (ii) Matsuda insulin sensitivity (Matsuda ISI) = 10000 / square root of [(FPG*FPI, fasting plasma insulin) * (mean OGTT plasma glucose*mean OGTT plasma insulin)], according to [22], (iii) insulinogenic index (IGI) for insulin secretion = (Ins30-Ins0)/(Glc30-Glc0) [23] and (iv) disposition index (Matsuda ISI * insulinogenic index). These parameters are derived from plasma glucose and plasma insulin concentrations, both, in basal state and during OGTT, and provide an estimate of insulin sensitivity and secretion. The frequently used HOMA-IR uses fasting glucose and insulin levels and is basically a marker of hepatic insulin sensitivity. In contrast, the Matsuda ISI, obtained from OGTT, reflects both, hepatic and peripheral insulin sensitivity [23]. For further analysis we only employed indices calculated from OGTT instead of single point fasting values, since these provide more accuracy.

Data are given as median [range] or unadjusted mean ± SEM. Not normally distributed parameters were log transformed to approximate normal distribution prior to statistical analysis. Differences in glucose metabolism between groups as well as factors affecting glucose metabolism were tested using multivariate linear regression model after adjustment for gender, age and body mass index (BMI). Results with values of *p* ≤ 0.05 were considered statistically significant. Receiver operating characteristics (ROC) analysis was performed for identification of cut-offs and the relative risk for the prevalence of prediabetes between groups was calculated. The JMP 10.0 (SAS Institute, Cary, NC) statistical software package was used.

## Results

### Patient characteristics

At time of investigation, 146 patients were on active waiting list of the Tübingen University Hospital Collaborative Transplant Center. Eight patients were excluded for not fasting. Characteristics of the 138 patients included are shown in *Table 1*. 114 patients were on hemodialysis (HD), 22 on peritoneal dialysis (PD) and 2 patients listed preemptively in the setting of combined transplantation. Median waiting time was 4.1 (0–13.3) years. 109 patients were listed for first transplantation, 29 for second or higher. 49 patients had a positive family history of DM in primary relatives, 2 patients a history of gestational diabetes.

**Table 1:** Patient characteristics.

General	
gender (f/m)	58 / 80
age (yrs)	51 [19–76]
dialysis modality (HD^1^ / PD^2^ / preemptive)	114 / 22 / 2
waiting time (yrs)	4.1 [0–13.3]
listed for kidney / SPK^3^	132 / 6
# of transplantation	
1^st^	109
2^nd^ or more	29
Metabolic	
Family history for DM^4^ (*n*)	49
gestational DM^4^ (*n*)	2
BMI^5^ (kg/m^2^)	25.6 [16.7–38.2]
FPG^6^ (mmol/l)	4.9 [3.1–15.8]
HbA1c^7^ (%)	5.5 [4.3–8.6]
total cholesterol (mmol/l)	4.9 [2.1–9.0]
LDL^8^ cholesterol (mmol/l)	2.2 [0.6–5.2]

1) hemodialysis

2) peritoneal dialysis

3) simultaneous pancreas-kidney transplantation

4) diabetes mellitus

5) body mass index

6) fasting plasma glucose

7) glycated hemoglobin A1c

8) low density lipoprotein.

Causes of end stage renal disease were glomerulonephritis (n, 54), hypertensive nephropathy (10), autosomal-dominant polycystic kidney disease (16), hereditary nephropathy (6), vesicourethral reflux (9), atrophic kidneys (6) and unknown or other (12). In 25 patients, diabetic nephropathy was the cause of end stage renal disease.

### Prevalence of diabetes and prediabetes

**Fig 1:**
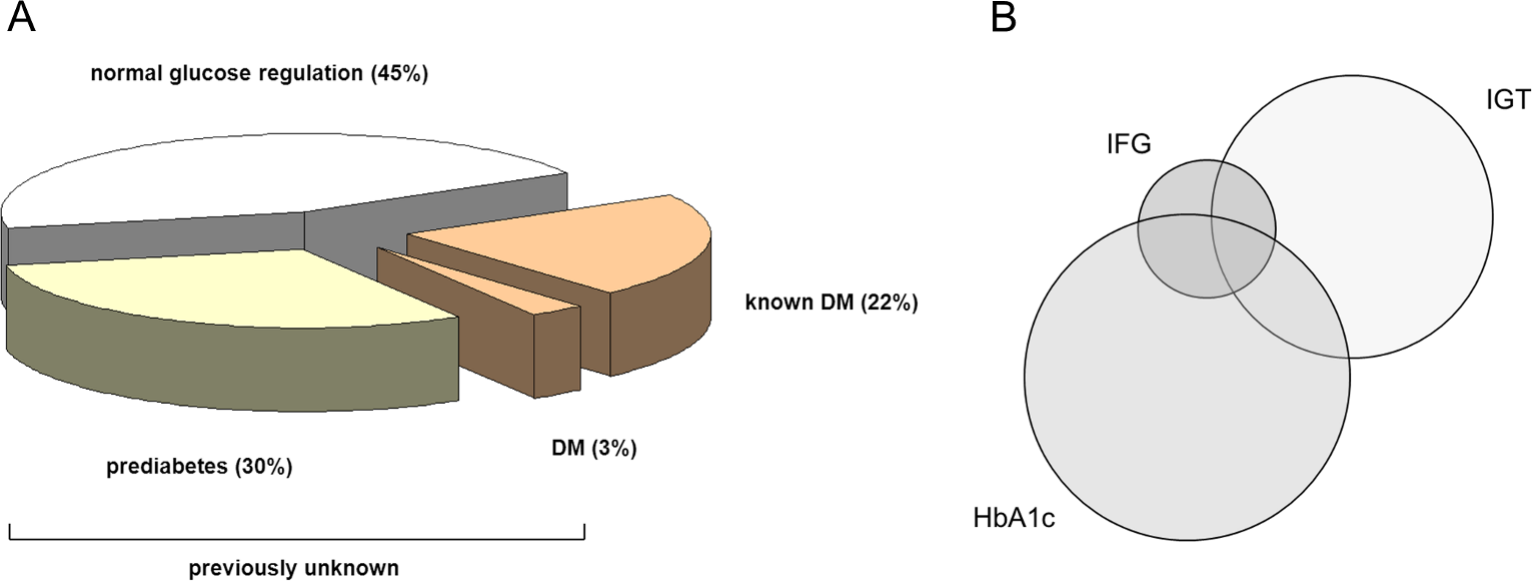
A, B: Prevalence of disturbances in glucose metabolism. **A**: Prevalence of known and previously unknown disturbances in glucose metabolism in waiting list patients (*n* = 138), DM: diabetes mellitus. **B**: Diagnostic criteria in patients diagnosed with prediabetes (*n* = 42). 10 patients fulfilled more than one criterion.

Of investigated patients, 30 (22%) had known diabetes mellitus; 14 with type 1 DM (among them 6 listed for simultaneous pancreas-kidney transplantation), and 16 with type 2 DM. 4 patients (3%) were newly diagnosed with manifest DM according to ADA criteria. 42 patients (30%) were detected to have prediabetes *(Fig 1A).* Of these, 7 patients showed IFG, 20 patients had IGT and 27 had an HbA1c between 5.7–6.4%, whereby 10 patients fulfilled more than one diagnostic criterion *(Fig 1B).* Overall, more than half of patients on active waitlist (55%) showed disturbances in glucose metabolism.

### Metabolic phenotyping

**Fig 2:**
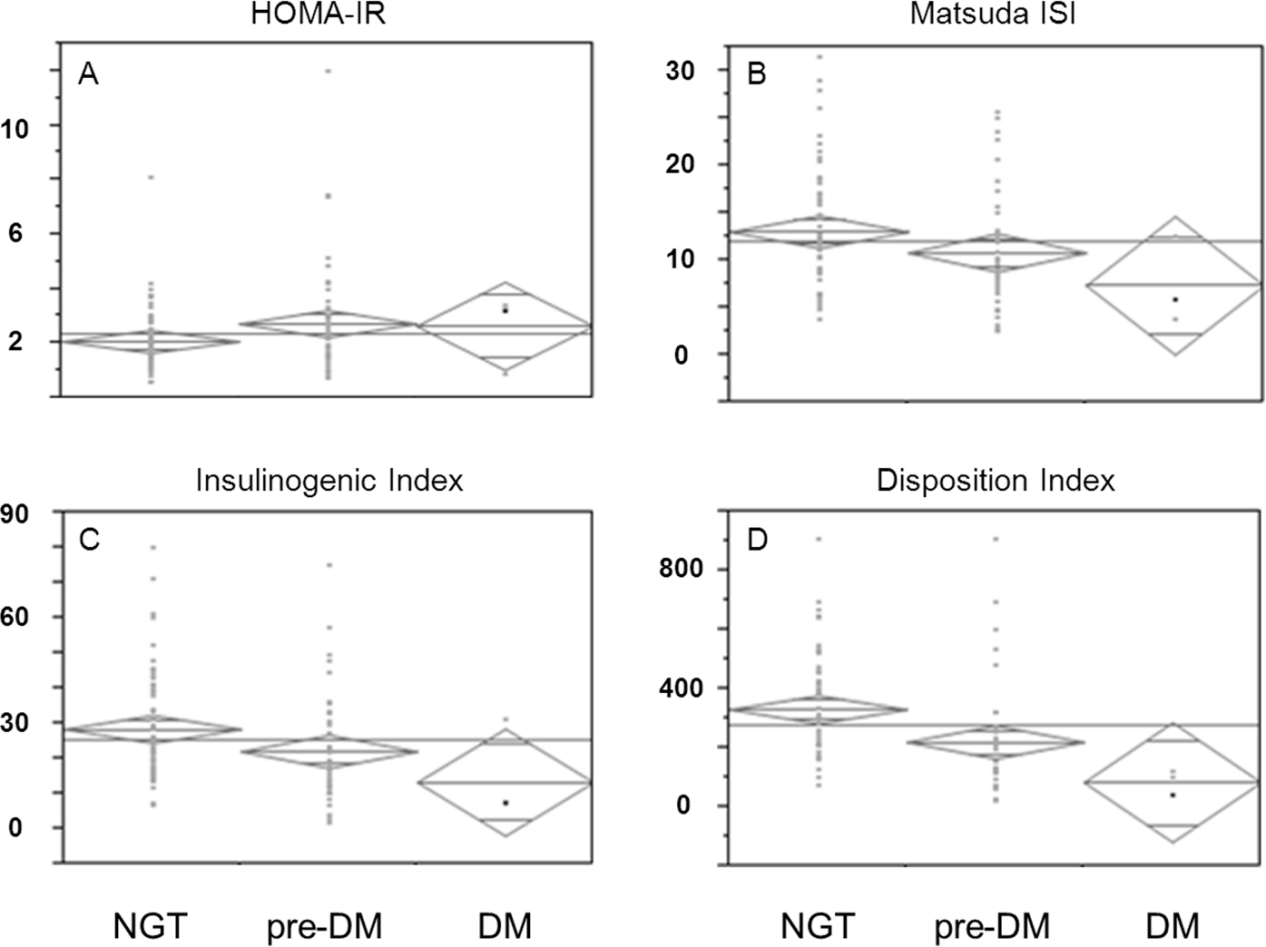
A—D: Insulin sensitivity and-secretion in all patients according to glucose metabolism. Descriptive illustration of insulin sensitivity and-secretion in patients with normal glucose tolerance (NGT), prediabetes and diabetes mellitus (DM). **A**: insulin sensitivity estimated by HOMA-IR; **B**: insulin sensitivity estimated by Matsuda ISI; **C**: insulin secretion estimated by insulinongenic index; **D**: insulin secretion in relation to insulin sensitivity, estimated by disposition index.

For in depth analyses, OGTT-derived metabolic parameters were assessed for characterization of underlying pathomechanism in patients without known DM. HOMA-IR and Matsuda ISI indices were used for estimation of insulin sensitivity and insulinogenic index for insulin secretion, respectively. The disposition index sets insulin secretion in relation to insulin sensitivity. Compared to patients with normal glucose tolerance (NGT), insulin sensitivity was lower and insulin secretion impaired in patients with disturbances in glucose metabolism such as prediabetes or newly diagnosed diabetes mellitus (*Fig 2A–2D*).

**Fig 3:**
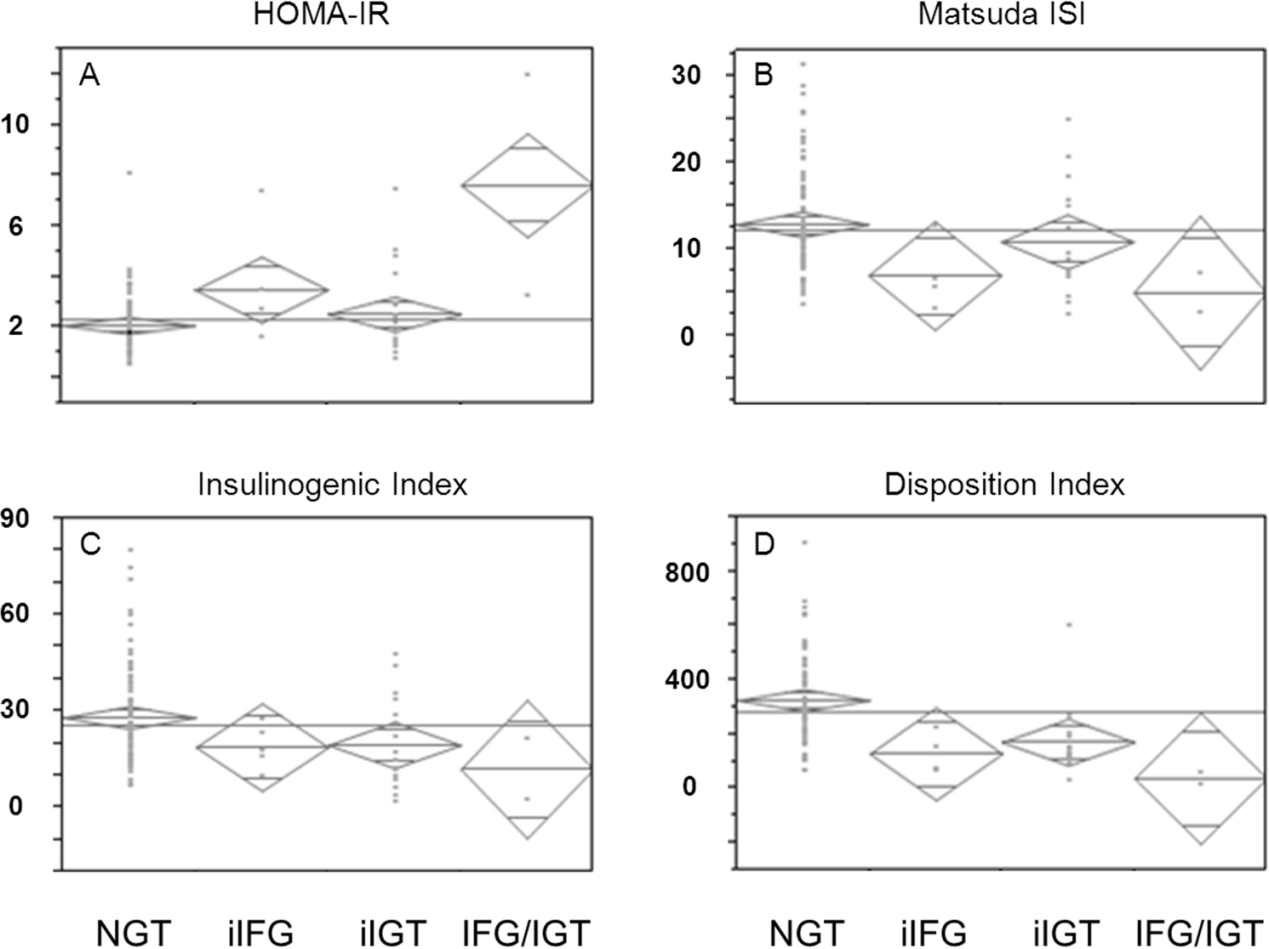
A—D: Insulin sensitivity and-secretion in patients without DM according to glucose tolerance status. Differences in insulin sensitivity and-secretion in patients without manifest DM (n = 104), as by normal glucose tolerance (NGT), isolated impaired fasting glucose (iIFG), isolated impaired glucose tolerance (iIGT) and both (IFG/IGT); **A**: insulin sensitivity estimated by HOMA-IR; **B**: insulin sensitivity estimated by Matsuda ISI; **C**: insulin secretion estimated by insulinongenic index; **D**: insulin secretion in relation to insulin sensitivity, estimated by disposition index.

To delineate underlying pathomechanisms, patients were grouped into normal glucose tolerance (NGT), isolated IFG (iIFG), isolated IGT (iIGT) or both (IFG/IGT). Insulin sensitivity was markedly impaired in patients were IFG was present (iIFG or IFG/IGT), using both, HOMA-IR and Matsuda ISI for estimation (*Fig 3A and 3B*), with *p* = 0.01 for HOMA-IR and *p* = 0.01 for Matsuda ISI, respectively, after adjustment for gender, age and BMI. Insulin secretion was impaired in iIFG, iIGT and most in IFG/IGT using insulinogenic index for estimation (*Fig 3C* *; p* = 0.02, adjusted for gender, age and BMI). The disposition index, as an overall risk marker was substantially worse if iIFG or iIGT or, especially, if both were present (*Fig 3D; p* < 0.001 after adjustment for gender, age and BMI).

### Factors affecting insulin sensitivity and-secretion

Multivariate analysis was used to determine factors affecting metabolic phenotype (*Table 2*). Age was a strong independent risk factor affecting insulin secretion (*p* < 0.001) and remained statistically significant when set in relation to insulin sensitivity (disposition index: *p* = 0.01). Higher BMI was shown to be an independent risk factor for decreased insulin sensitivity (*p* < 0.001). However, this was in part compensated by higher insulin secretion (*p* = 0.04), thereby rendering the effect on disposition index smaller (*p* = 0.04). Gender, family history for DM, dialysis modality, waiting time and previous transplantation were no independent risk factors for disturbed glucose metabolism in our cohort (*Table 2*).

**Table 2:** Factors independently affecting glucose metabolism.

	Matsuda ISI ^1^			IGI ^2^			disposition index		
	*ß*	*p*	95% CI	*ß*	*p*	95% CI	*ß*	*p*	95% CI
gender	0.09	0.29	-0.05 to 0.15	0.03	0.80	-0.12 to 0.15	0.09	0.33	-0.07 to 0.21
age	0.06	0.54	-0.28 to 0.53	-0.40	**<0.001** *	-1.58 to -0.46	-0.33	**0.01** *	-1.48 to -0.31
BMI ^3^	-0.58	**<0.001** *	-2.79 to -1.37	0.23	**0.04** *	0.02 to 2.00	-0.23	**0.04** *	-2.10 to -0.04
FH of DM ^4^	-0.003	0.9	-0.11 to 0.10	0.19	0.08	-0.02 to 0.29	0.17	0.10	-0.03 to 0.29
dialysis modality	-0.09	0.30	-0.20 to 0.06	0.01	0.89	-0.17 to 0.19	-0.06	0.56	-0.24 to 0.13
waiting time	-0.05	0.56	-0.19 to 0.10	0.17	0.09	-0.03 to 0.38	0.12	0.22	-0.08 to 0.34
1^st^ vs. re-tx	0.005	0.9	-0.12 to 0.13	0.15	0.18	-0.05 to 0.29	0.15	0.18	-0-06 to 0–30

Multivariate linear regression analysis

* *p*-values ≤ 0.05 were considered statistically significant

1) Matsuda index for insulin sensitivity

2) insulinogenic index

3) body mass index

4) family history of diabetes mellitus.

### Clinical risk stratification

BMI and age were identified as independent influencing factors of metabolic phenotype. Accordingly, the prevalence of prediabetes increased with BMI. Using ROC analysis, BMI with a cut-off of 22.3 kg/m^2^ predicted the risk of being diagnosed with prediabetes (sensitivity 0.86, specificity of 0.45, AUC 0.56). In patients with a higher BMI, the relative risk of prevalent prediabetes was 1.93 (range 0.97–3.85) as compared to patients below this cut-off. In patients with prediabetes, insulin sensitivity was lower in patients with a BMI ≥ 22.3 kg/m^2^ than in patients below this cut-off (Matsuda ISI 10.17 ± 6.02 vs. 14.74 ± 6.25, *p* = 0.11 after adjustment for gender and age). Insulin secretion (IGI) showed no difference (*p* = 0.9).

**Fig 4:**
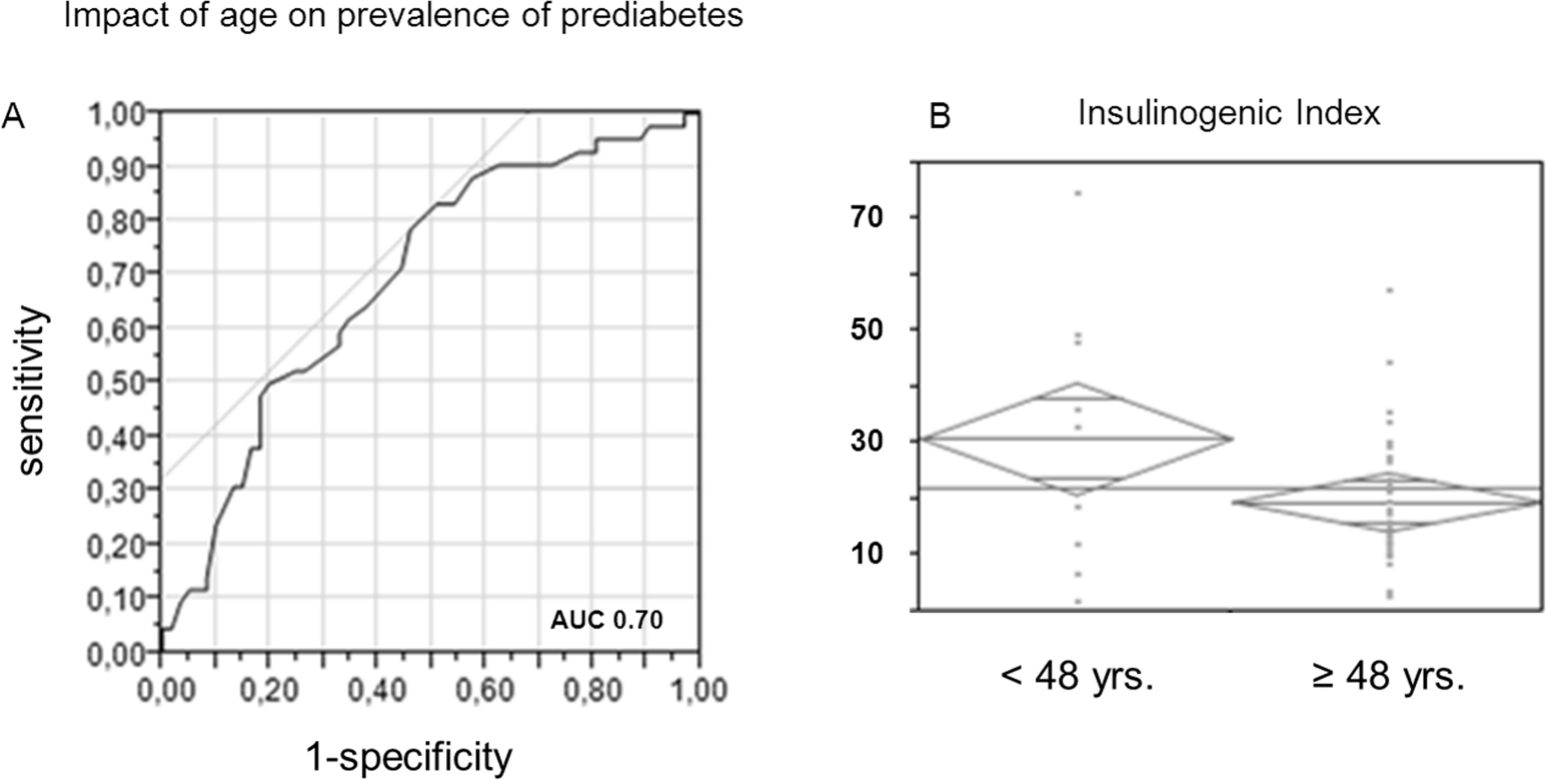
A, B: Influence of age on prevalence of prediabetes and insulin secretion. **A:** Receiver operation characteristics (ROC) analysis of age predicting the prevalence of prediabetes. **B**: Insulin secretion estimated by insulinongenic index in patients with prediabetes according to age cut-off.

For age, ROC analysis revealed an optimum cut-off to predict the risk of being diagnosed with prediabetes of 48 years (sensitivity 0.78, specificity 0.55, AUC 0.70, *Fig 4A*). In patients 48 years of age or older, the relative risk of being diagnosed with prediabetes was 2.59 (95% CI 1.38–4.83), as compared to younger patients. IGI, reflecting insulin secretion was lower in patients above this cut-off, compared to patients below (IGI 19.78 ± 11.57 vs. 31.06 ± 7.91, *p* = 0.35 after adjustment for gender and BMI) (*Fig 4B),* whereas insulin sensitivity showed no difference (Matsuda ISI: *p* = 0.55).

## Discussion

A principal finding of the present study is that among waiting list patients, disturbances in glucose metabolism are substantially higher than previously anticipated and published [24]. Using all diagnostic ADA criteria, we demonstrate these to be as high as 55% in patients on active kidney waiting list, with 33% of patients previously undiagnosed. These numbers are particularly impressive, since transplant candidates usually represent a carefully screened positive selection among dialysis patients. In such a general dialysis cohort, Krämer and coworkers [19] showed about the same overall prevalence, however with a higher proportion of known DM, supporting the notion that manifest DM limits access to the waiting list and transplantation. For waiting list patients, only few data exist, all without comprehensive characterization of glucose metabolism. Caillard and coworkers [24], in 234 waitlist candidates, found disturbances in glucose metabolism in 18.5% of previously undiagnosed patients, using IGT and manifest diabetes as criteria. This is probably underestimated and we demonstrate that, using the full diagnostic spectrum (fasting plasma glucose, OGTT, HbA1c), the prevalence is almost double. The role of OGTT is highlighted by the fact, that in our cohort, all patients with newly diagnosed diabetes mellitus were detected by a 2 hour plasma glucose concentration ≥ 200 mg/dl in OGTT, showing normal fasting plasma glucose and HbA1c values. Also, in patients with prediabetes, only about two thirds of patients would have been diagnosed by HbA1c and fasting plasma glucose alone, leaving one third of patients undiagnosed (cf. *Fig 1B*). For identification of patients at risk for progression to DM or PTDM, therefore, complete characterization of glucose metabolism is mandatory. This holds especially true, since the diagnostic accuracy of HbA1c alone in chronic kidney disease (CKD) and dialysis patients is limited, with several potential confounders described [25]. To minimize interference with analytics, high performance liquid chromatography was used for determination of HbA1c in our investigations. HbA1c values did not differ by dialysis modality in our cohort. Most studies report lower HbA1c values in dialysis patients due to higher erythrocyte turnover [26], such that the number of patients with diabetes or prediabetes on kidney transplant waiting list diagnosed via HbA1c may even be underestimated. On the other hand, those with elevated HbA1c are highly probable to have disturbances in glucose metabolism.

To our knowledge, we report for the first time a CKD V cohort on kidney transplant waiting list that has been fully metabolically phenotyped. We used OGTT with determination of plasma insulin levels, allowing individual assessment of insulin sensitivity and–secretion with higher accuracy than single point fasting values. The respective indices are established in diabetes research and allow delineating underlying pathomechanisms of disturbed glucose metabolism. This is essential for effective intervention and risk reduction.

Patients at maximum risk of progression to DM as well as PTDM are patients with prediabetes. In our cohort, one third of patients presented with prediabetes. Of note, prediabetes is not uniform. Individuals with impaired fasting glucose (IFG) had markedly lower insulin sensitivity, in accord with the fact that impaired insulin-induced suppression of gluconeogenesis due to hepatic insulin resistance leads to higher fasting plasma glucose [27]. Individuals with impaired glucose tolerance (IGT) showed significant lower insulin secretion. This reflects impaired insulin response to glucose challenge, resulting in higher plasma glucose concentration. IGT has been shown to imply a higher risk for progression to manifest DM than IFG [5], consistent with the notion that disturbances in insulin secretion rather than-sensitivity seem to be the principal underlying pathomechanism of DM. The latter also holds true for PTDM [12,16], albeit in the first year after kidney transplantation, insulin sensitivity may be the dominant cause [28]. It is currently unknown, whether IFG and IGT also differ regarding risk of progression to PTDM, which would allow for further risk stratification.

When looking at factors influencing glucose metabolism in our cohort, age and BMI independently affected insulin sensitivity and-secretion. Higher age was associated with a decrease in insulin secretion, reflecting normal aging process and apoptosis of beta cells. Consistently, higher age also predicted the prevalence of prediabetes in waiting list patients with a more a 2.5-fold relative risk of having prediabetes in patients over the age of 48 years. This affects a large proportion of patients waiting for a kidney transplant. In 2013, less than 35% of adult kidney transplant recipients in the U.S. were below 50 years of age [29]. In our cohort, only 38.4% of patients were younger than 48 years, with 61.6% at significantly elevated risk. Higher BMI strongly affected insulin sensitivity. When looking at prediabetes as clinical endpoint, the association was less strong. The optimum cut-off to predict prediabetes in our cohort was within the range of normal body weight. However, it is known that BMI affects insulin sensitivity as a continuous variable even within the normal weight range [30]. Also, we demonstrate that impaired insulin sensitivity is compensated in part by increased insulin secretion, weakening correlation of BMI to clinical endpoints. Taken together, the risk of prediabetes increases with age and BMI.

Based on knowledge about patients at risk and contributing pathophysiology, a number of preventive strategies can be discussed: For insulin secretion, protecting and improving beta cell function is essential. An option for protection of beta cell function is the perioperative administration of basal insulin, as has been shown by the working group of Säemann et al. [31]. Interestingly, one year after transplantation, the treatment group showed no difference in insulin sensitivity but markedly improved beta cell function, as measured by insulinogenic index, even though insulin administration had been stopped 3 months after transplantation. The protective effect of early insulin administration has also been shown in non transplant populations [32], and is an established concept also in pancreas and islet cell transplantation [33,34]. An intriguing novel concept is the beta cell protective effect of incretins [35,36]. In pancreatic islet cell transplantation, incretins have been used to improve clinical outcome [37], and recently, the protective effect on beta cells has been shown in this setting [38]. The use of incretin analogues and dipeptidyl peptidase (DPP)-4 inhibitors is not yet established in solid organ transplantation. Potential limitations include altered adsorption of immunosuppression, although a recently published small case series indicates that coadministration of the incretin analogue liraglutide with tacrolimus may be safe [39]. Similar results have been shown in a pilot study by Lane and coworkers, using DPP-4 inhibitors [40]. Importantly, both incretin analogues and DPP-4 inhibitors carry no risk of hypoglycemia; the benefit of the latter is that DPP-4 inhibitors can safely be given irrespective of renal function, including dialysis. As to the controversy regarding DPP-4 inhibitors and associated cardiovascular risk, an increased rate of hospitalization for heart failure has been reported [41], whereas no increased rate of cardiovascular events was found in others [42–44], including the very recently published TECOS study with 14,671 patients. Hence, protecting the beta cell by insulin or incretin action seems a feasible approach in patients at risk for beta cell failure.

For insulin sensitivity, body weight is a principal modifiable factor. We demonstrate in our cohort, that among overweight waiting list patients, impaired insulin sensitivity is at large compensated by increased insulin secretion (cf. Table 2). However, in situations when other risk factors, i.e. immunosuppression, add to obesity, improving insulin sensitivity may be of help. Lifestyle intervention and reducing body weight is a preventive strategy. Such preconditioning on waiting list may be highly effective in improving glucose metabolism. In a large cohort, lifestyle intervention in prediabetic patients reduced the incidence of DM diabetes by 58% [45], even if normalisation of glucose metabolism was transient [46]. Of note, in patients on dialysis, weight loss is not uniformly beneficial. Mortality in dialysis patients was shown to be lower in patients with higher BMI, even in higher grades of obesity [47]. This so-called reverse epidemiology may prevent physicians to impose too strictly on dialysis patients to loose weight. However, not only metabolic disturbances but also periinterventional complications such as wound healing, deep vein thrombosis and delayed graft function are more frequent in obese renal allograft recipients [48]. Therefore, in obese patients aiming for kidney transplantation, a regression to normal body weight seems reasonable.

Early identification of patients at risk and knowledge of underlying pathomechanisms may also help tailoring immunosuppression. Immunosuppression is a major modifiable variable for glycemic control, as for the risk of allograft rejection, infection and malignancy. It has to be made very clear, that PTDM is an important but not the only aspect in individualized immunosuppression. Accordingly, a recent consensus statement on PTDM sounded a note of caution regarding changes in immunosuppression in patients with PTDM and especially in patients at risk alone [49].

Basically, improvement of glucose metabolism has been shown for ciclosporine, as compared to tacrolimus-based regimen [50], including reversal of manifest PTDM [51]. The role of corticosteroid avoidance or withdrawal is much more controversial: It has been shown that early withdrawal of corticosteroids is effective in preventing PTDM [52], however, a meta-analysis on steroid withdrawal between 3 and 6 months after transplantation found no effect on PTDM [53]. Mycophenolate mofetil (MMF) does not seem to have an effect on glucose metabolism [54]; one study reported a lower incidence of PTDM with the use of MMF, however that effect was attributed to the lower dose of CNI and corticosteroids in combination with MMF [9].

Based on our findings, selected patients with insulin resistance, i.e. overweight patients, may nonetheless benefit from steroid-free maintenance immunosuppression, since corticosteroids negatively affect insulin sensitivity. At our center, corticosteroid-free maintenance immunosuppression is used for all standard immunological risk patients with excellent long-term allograft survival. Calcineurin inhibitors (CNI) have been shown to directly impair beta-cell function [55]. Therefore, patients with impaired insulin secretion such as patients of higher age may benefit from a low-dose CNI or CNI-free regimen, if the immunological situation allows for it. In any case, careful outweighing of immunological and non-immunulogical risk is key for determining individual immunosuppression.

The present study does have limitations. Firstly, as a single center analysis, our cohort is restricted to the number of patients on active kidney transplant waiting list. Nonetheless, our work is the first to thoroughly characterize a complete waiting list and patients with prediabetes, i.e. those of primary interest, account for a sufficiently large proportion. A second limitation is the homogeneity of the cohort, with patients almost all of European descent. We are fully aware, that differences in ethnicity affect glucose metabolism, however addressing this issue is beyond the scope of this investigation. Lastly, our study is a cross-sectional approach; however, it provides a comprehensive basis for interventional trials in waiting list patients.

In summary, the present investigations advance current knowledge in a number of aspects. The number of patients at risk is by far higher than currently anticipated. Screening without OGTT will leave 30% of patients undiagnosed. Metabolic phenotyping helps identifying patients at risk. In settings where assessment of insulin sensitivity and-secretion is not readily available, we demonstrate an approach to guide therapy. Our data provide a basis for early individual risk stratification and specific intervention to improve patient and allograft outcome.
